# Managing uncertain recovery for patients nearing the end of life in hospital: a mixed-methods feasibility cluster randomised controlled trial of the AMBER care bundle

**DOI:** 10.1186/s13063-019-3612-0

**Published:** 2019-08-16

**Authors:** J. Koffman, E. Yorganci, D. Yi, W. Gao, F. Murtagh, A. Pickles, S. Barclay, H. Johnson, R. Wilson, L. Sampson, J. Droney, M. Farquhar, T. Prevost, C. J. Evans

**Affiliations:** 1 King’s College London, Cicely Saunders Institute, London, UK; 2 Wolfson Palliative Care Research Centre, Hull York Medical School, University of Hull, Hull, UK; 3 King’s College London, Clinical Trials Unit, London, UK; 4 Primary Care Unit, Department of Public Health and Primary Care Organisation, University of Cambridge, Cambridge, UK; 5 Marie Curie Palliative Care Research Department, University College London, London, UK; 6 The Royal Marsden NHS Foundation Trust, London, UK; 7 School of Health Sciences, Faculty of Medicine and Health, University of East Anglia, Norwich, UK; 8 Imperial College London, Imperial Clinical Trials Unit, School of Public Health, London, UK; 9 Sussex Community NHS Foundation Trust, Brighton General Hospital, Brighton, UK

**Keywords:** Feasibility study, clinical trial, mixed methods, palliative care, end-of-life care, clinical uncertainty, AMBER care bundle, complex intervention

## Abstract

**Background:**

The AMBER (Assessment, Management, Best Practice, Engagement, Recovery Uncertain) care bundle is a complex intervention used in UK hospitals to support patients with uncertain recovery. However, it has yet to be evaluated in a randomised controlled trial (RCT) to identify potential benefits or harms. The aim of this trial was to investigate the feasibility of a cluster RCT of the AMBER care bundle.

**Methods:**

This is a prospective mixed-methods feasibility cluster RCT. Quantitative data collected from patients (or proxies if patients lack capacity) were used (i) to examine recruitment, retention and follow-up rates; (ii) to test data collection tools for the trial and determine their optimum timing; (iii) to test methods to identify the use of financial resources; and (iv) to explore the acceptability of study procedures for health professionals and patients. Descriptive statistical analyses and thematic analysis used the framework approach.

**Results:**

In total, 894 patients were screened, of whom 220 were eligible and 19 of those eligible (8.6%) declined to participate. Recruitment to the control arm was challenging. Of the 728 patients screened for that arm, 647 (88.9%) were excluded. Overall, 65 patients were recruited (81.3% of the recruitment target of 80). Overall, many were elderly (≥80 years, 46.2%, *n* = 30, mean = 77.8 years, standard deviation [SD] = 12.3 years). Over half (53.8%) had a non-cancer diagnosis, with a mean of 2.3 co-morbidities; 24.6% patients (*n* = 16) died during their hospital stay and 35.4% (*n* = 23) within 100 days of discharge. In both trial arms, baseline IPOS subscale scores identified moderate patient anxiety (control: mean 13.3, SD 4.8; intervention: mean 13.3, SD 5.1), and howRwe identified a good care experience (control: mean 13.1, SD 2.5; intervention: mean 11.5, SD 2.1). Collecting quantitative service use and quality of life data was feasible. No patient participants regarded study involvement negatively. Focus groups with health professionals identified concerns regarding (i) the subjectivity of the intervention’s eligibility criteria, (ii) the need to prognosticate to identify potential patients and (iii) consent procedures and the length of the questionnaire.

**Conclusions:**

A full trial of the AMBER care bundle is technically feasible but impractical due to fundamental issues in operationalising the intervention’s eligibility criteria, which prevents optimal recruitment. Since this complex intervention continues to be used in clinical care and advocated in policy, alternative research approaches must be considered and tested.

**Trial registration:**

International Standard Randomised Controlled Trial Number (ISRCTN) Register, ISRCTN36040085.

## Background

### Clinical uncertainty in hospital settings

Clinical uncertainty has been defined as the inability to determine the meaning and significance of illness-related events [1]. It occurs when health professionals are unable to predict outcomes accurately due to insufficient information. Evidence suggests that in the last 30 days of life, the combination of deteriorating health and clinical uncertainty is highly distressing for patients in hospital and their families [2, 3]. This is amplified when discussions about their situation and preferences for care and location of death do not occur. Most people (67–80%) want to be informed about their poor prognosis [4]. Research, however, has identified that discussions about prognosis rarely occur [5]. This increases the likelihood of hospital deaths and also leads to poor care satisfaction, mistrust and loss of confidence in health professionals [6–9] and may lead to complaints [10]. Clinical uncertainty also impacts the clinicians’ confidence and their practice. Health professionals frequently struggle with uncertainty, which can result in overtreatment or over-investigation [11], lack of communication with patients about their future [12, 13], and increased care costs [14].

### The potential for better care and the AMBER care bundle

In recent years, complex interventions [15, 16] aimed at improving the care of patients who may be approaching the end of life have become more common [17, 18]. In 2010, the AMBER care bundle was developed to improve care in the acute hospital setting for patients who are deteriorating, clinically unstable with limited reversibility, and at risk of dying in the next 1 to 2 months [19]. This was subsequently amended to be at risk of dying during their current episode of care despite treatment. The algorithmic intervention of the AMBER care bundle is designed to encourage health professionals to work with patients and families to develop and document a clear medical plan, including consideration of anticipated outcomes, cardiopulmonary resuscitation and escalation status, while acknowledging the uncertainty. This plan is revisited daily and encourages regular communication with the patient and their family regarding treatment plans, place of care and any other concerns. The bundle was designed to work alongside active medical care when uncertainty about the outcome remains.

A recent non-randomised comparative study of the AMBER care bundle with standard care, conducted in the UK, identified mixed findings. In comparison to similar patients in the control group, the use of the AMBER care bundle was associated with shorter lengths of hospital stay, more frequent discussions about prognosis between health professionals and patients, and higher awareness by patients of their prognosis. The clarity of the information provided, however, was rated lower by this group compared to those in the control group [20]. Qualitative research among health professionals has identified that the AMBER care bundle was often used as a tool to label or categorise patients, and indirectly served a symbolic purpose in affecting the behaviour of individuals and teams. Participants described the importance of the training associated with the intervention but reported that adequate exposure to the intervention, and the learning, varied [21].

Clinical equipoise, therefore, still exists in relation to the AMBER care bundle. A robust comparative evaluation of the intervention compared to standard care is, therefore, needed. The UK Medical Research Council’s guidance on the development and evaluation of complex interventions [15] and the Methods of Researching End-of-life Care (MORECare) statement [16] both recommend a feasibility study before full evaluation. Feasibility studies, now more common in palliative and end of life care [22], enable researchers to identify problems that might undermine the acceptability and delivery of the intervention or the conduct of a fully powered trial [23, 24]. Researchers are then potentially able to remedy problems with the intervention, trial design, or conduct by returning to the development phase, rather than proceeding to a full trial. This has important implications for the efficient use of resources, ensuring they are not directed to studies that produce a null result due to an unfeasible study design [25]. Moreover, it is also unethical to run a full trial before running a feasibility study.

In this paper, we report on the feasibility of conducting a pragmatic multi-centre cluster randomised controlled trial (RCT) of a hospital-based complex intervention (the AMBER care bundle) that aims to better serve patients whose situations are clinically uncertain and where there is a risk that they will die during their hospital stay, versus standard care. Four feasibility objectives were specified:

1. To examine recruitment, retention, and follow-up rates
2. To test data collection tools for the trial and determine what would be their optimum timing in a larger trial
3. To test methods for identifying the use of financial resources
4. To explore the acceptability of study procedures for patients and health professionals.

## Methods

### Design

This study was registered with the International Standard Randomised Controlled Trials registry (ISRCTN: 36040085). Favourable ethical opinions were obtained from the national research ethics committee for Camden and King’s Cross (16/LO/2010) and the Health Research Authority. National Health Service (NHS) research governance approvals were obtained from each participating study hospital.

We conducted a parallel cluster RCT with a 1:1 allocation ratio, employing convergent mixed methods, with the quantitative and qualitative data given equal importance. Data were collected sequentially and analysed concurrently. A cluster RCT design was chosen because the implementation and delivery of the intervention were at an organisational level, in this case, hospital wards, and not the patients. Cluster RCTs are used to avoid contamination between treatment groups [26, 27]. This study comprised the trial, an examination of patients’ clinical records, and focus groups with health professionals.

### Study setting

The study took place across purposefully selected general medical wards in four clusters, in this case, district general hospitals (DGHs) in England. DGHs are major secondary-care facilities that typically provide an array of diagnostic and therapeutic services to the local population. There are over 142 DGHs in the UK [28]. The four DGHs selected serve diverse populations, including those with ethnic diversity and material deprivation. The hospitals have different strengths and weaknesses in terms of their Care Quality Commission ratings [29].

Participant recruitment and implementation of the AMBER care bundle were limited to one or two general medical wards at each hospital site. Selection of study wards at each site was informed by heat maps that provided contextual information at a ward level on the number of deaths during and up to 100 days after admission. Additional data comprised the number of patients who died with an individualised approach to the last days of life care and the number of hospital readmissions prior to a patient’s death. Wards with the highest number of deaths per year were considered to be suitable for the study.

### Randomisation and masking

Hospitals were randomly assigned to the intervention or control arms at the level of the cluster via an independent clinical trials unit by randomly sequencing the order of randomisation and then randomising the sites in this order into fixed blocks of two. Research nurses collecting data from patient participants were not masked to the group allocation. Quantitative analyses were performed, masked for the group allocation.

### Patient participants and the recruitment process

Research nurses identified patients (or their proxies) in the intervention and control wards daily who fulfilled the eligibility criteria aligned with those of the AMBER care bundle i.e. patients:

1. who were deteriorating, whose status was clinically uncertain, and with limited reversibility
2. at risk of dying during their current episode of care, despite treatment
3. able to provide written informed consent or where a personal consultee could be identified and approached to give an opinion on whether the patient would have wished to participate in the study.

Research nurses scanned the hospital ward whiteboards to identify potential patient participants, who were then discussed with the clinicians to confirm their suitability for the study. All participants were considered to have mental capacity unless this was established otherwise, and all practicable steps were taken to enable individuals to decide for themselves whether they wished to participate. Potential participants’ level of capacity was discussed with referring clinicians to identify those with possible impaired capacity and to anticipate the likely consent procedure. Capacity was established in the initial meeting with the patient using the Mental Capacity Act (MCA) four-step process [30]. It was assessed whether the individual can:

(i) understand the information given to them about the study
(ii) retain the information (even for a short time)
(iii) use or evaluate that information
(iv) communicate their decision (by any means).

### Recruitment of staff members to focus groups

Health professionals from study wards and the research nurses were invited via posters to participate in one of the four focus groups representing the study wards. Of those who expressed interest, we attempted to recruit a range of health professionals with different levels of experience. Written informed consent was obtained from health professionals prior to the focus groups taking place. Consent was obtained at the end of the focus groups from any health professionals who joined late but wished to participate.

### Data collection

#### Patient (or proxy) questionnaires and outcome measures

After obtaining informed consent or, for adults lacking capacity, permission from a proxy (A third party), research nurses conducted baseline face-to-face interviews with patient participants, or their proxies, on the study wards. A questionnaire captured patient participant demographic and clinical information. Health performance status was assessed using the Australia-modified Karnofsky Performance Scale [31] and the following measures.

#### Patient and family anxiety and communication

The first of the two candidate primary outcome measures was the patient/family anxiety and communication subscale of the Integrated Palliative care Outcome Scale (IPOS) [32, 33]. These data were collected at baseline, 3–5 days, and 10–15 days. This patient-centred outcome was chosen because of the intended benefits of the AMBER care bundle and the findings from a comparative observational study in which psychosocial issues were identified as a central concern to patients and their families [34]. The patient/family anxiety and communication subscale includes items on (i) being in receipt of information, (ii) addressing practical matters, (iii) sharing feelings with family, (iv) being at peace, (v) the patients’ level of anxiety and depression, and (vi) family distress and ability to share feelings.

#### Patient experience

The second candidate primary outcome measure was howRwe [35], a patient-reported experience measure that examines changes in patients’ reported experiences of care. This was collected at baseline, 3–5 days, and 10–15 days. The measure, used among patients who possessed mental capacity, is succinct, comprising just four items relating to the delivery and organisation of care. howRwe has been used successfully for hospital inpatients, hospital outpatients, and general practice patients, and patients in care homes or domiciliary care [36, 37].

#### Health-related quality of life and health resource utilisation

The EQ-5D-5L [38] was used to measure health-related quality of life. It measures five health-related quality of life dimensions: (i) mobility, (ii) self-care, (iii) usual activities, (iv) pain and discomfort, and (v) anxiety and depression, using two descriptive systems and a visual analogue scale. These data were collected at baseline, 3–5 days, and 10–15 days. Each dimension has five levels, ranging from having ‘no problems’ to ‘being unable to perform’. The visual analogue scale records the respondent’s self-rated health on a vertical scale, where the endpoints are labelled ‘best imaginable health state’ and ‘worst imaginable health state’. This information can be used as a quantitative measure of health outcomes as judged by individual respondents.

Data on health resource utilisation were collected using the Client Service Receipt Inventory [39, 40]. These data were collected at baseline and 10–15 days. This inventory measures the use of health, social, and informal care in the 3 months prior to hospital admission, and then during the inpatient stay for up to 10–15 days (time point three).

#### Views on being involved in the study

Patient participants were asked to provide responses to questions about being involved in the study on a five-point scale ranging from 1 (highly positive) to 5 (highly negative). These views were collected at baseline, 3–5 days, and 10–15 days. In addition, they were asked if they would recommend or not recommend involvement in the study to other patients. This included an option for them to respond as ‘don’t know’ [41]. Free-text comments were also invited.

### Quantitative data analysis

**Fig. 1:**
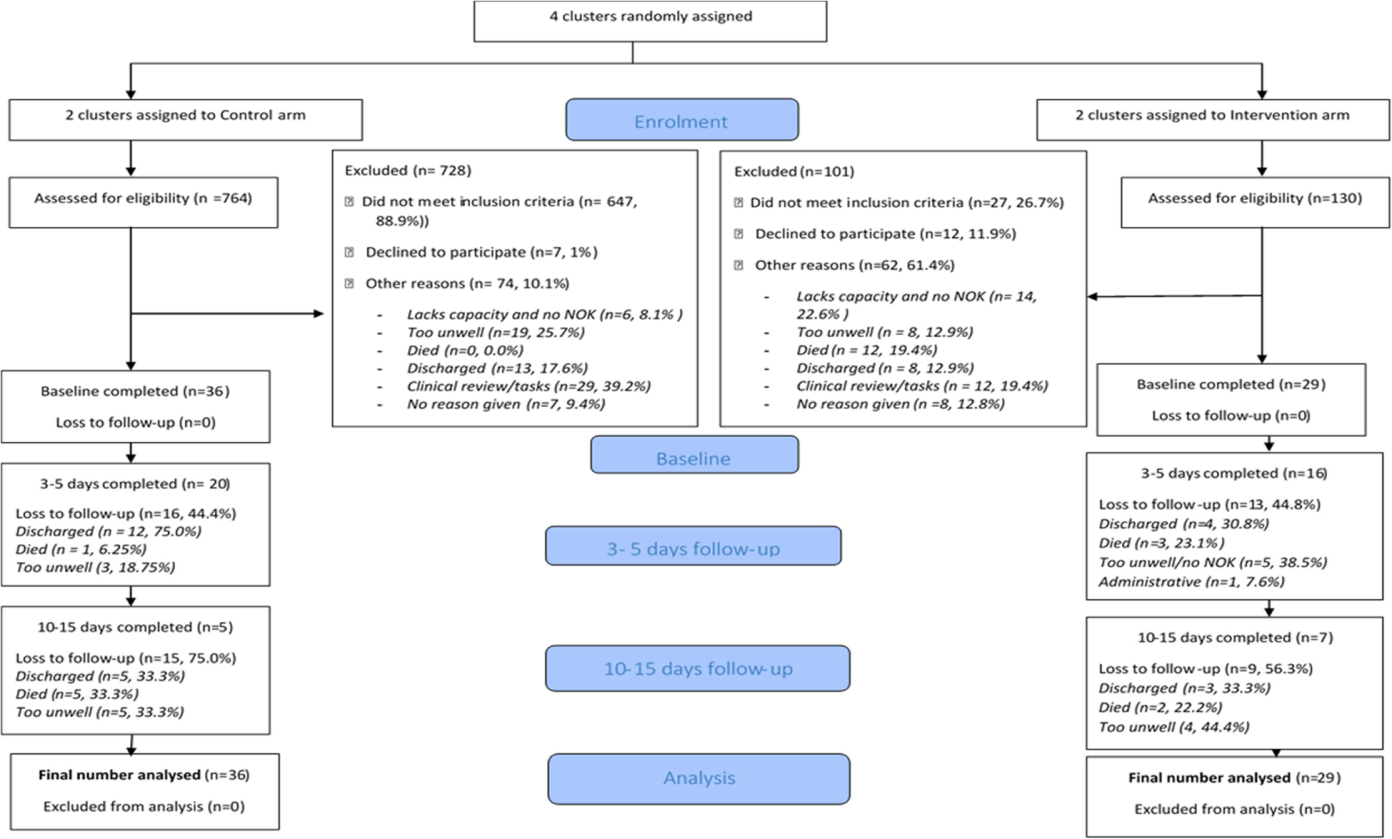
Consolidated Standards of Reporting Trial 2010 (CONSORT) flow diagram of study

The analysis followed the Consolidated Standards of Reporting Trials (CONSORT) guidelines (Fig. 1) and was conducted in collaboration with the clinical trials unit. Two statisticians (WG and RW), the chief investigator (JK), and the health economist (DY) were blind to the randomisation. Data were entered into predesigned Epidata databases [42]. In total 10% of the data were double entered and cross-checks were conducted. No discordance was detected for the candidate primary outcome measures (100% match for the IPOS subscale and howRwe) and very high accuracy for the rest of the questionnaires.

Since this was a feasibility study, a formal power calculation was not appropriate. Based on the information about the number of deaths and prior studies, we aimed to recruit 40 patients per study arm to provide us with sufficient data to test data collection forms and questionnaires, examine the appropriateness of candidate primary outcome measures, determine what would be the optimum data collection timing for a larger trial, and explore the acceptability of study procedures to patient participants. Any investigations of changes in study parameters were exploratory only.

Descriptive statistics on demographic and study variables were calculated as means, medians, ranges, standard deviations (SDs), and percentages (for categorical variables). No tests of significance were conducted. However, 95% confidence intervals, rounded up to one decimal place, were provided to indicate the precision of the estimates from the feasibility trial. The analysis of the IPOS data focused only on those participating patients with complete data for all IPOS patient and family anxiety and communication subscale items.

Economic evaluation is an emergent area in palliative care and ambiguity still surrounds best practice [39]. Procedures to inform the economic evaluation in the full cluster RCT protocol were reported, focusing on resource implications from health and social care, and societal perspectives. We aimed to make preliminary cost-effectiveness calculations (e.g. combining Client Service Receipt Inventory data on costs and the EQ-5D-5L score).

Responses to the five items in EQ-5D-5L were used to generate the index score for each patient. Theoretically, the index score ranges from 0 (death) to 1 (full health). Some EQ-5D-5L profiles were evaluated as below zero, implying that the individual considers their current quality of life as worse than death.

### Qualitative data

#### Focus groups

A topic guide was developed to explore health professionals’ views on the conduct of the feasibility cluster RCT. The four focus groups were led by two senior researchers (JK and CE), both of whom have experience in palliative care research and qualitative research. Field notes were taken by EY and HJ to provide a contextual understanding of any non-verbal communication expressed during the focus groups. All focus groups were audio-recorded and lasted 50 mins in intervention site 1, 49 mins in intervention site 2, 60 mins in control site 1, and 65 mins in control site 2.

#### Qualitative data analysis

The qualitative data analysis was informed by the framework approach, in which data are inductively coded and organised to identify themes emerging from the focus groups [43]. To address issues of rigour and trustworthiness in the analysis, we (JK, EY, and HJ) independently examined the focus group transcripts, met to develop a thematic framework, and then independently coded the transcripts. Where coding differed, these issues were reconsidered by JK, EY, and HJ in detail until a consensus was achieved [44]. To avoid making unwarranted claims about patterns and regularities in the data, care was taken to examine what appeared to be more unusual or non-confirmatory views and we considered what the data told us about their causes [44]. Excerpts from the focus group transcripts are presented to illustrate themes and represent a range of views, rather than being reliant on selected individuals. All quotes from health professionals have been anonymised to preserve confidentiality.

## Results

### Recruitment, retention and follow-up rates

We had initially planned for recruitment to take place at each of the study sites for 3 months, with an expected average of seven participants being consented per month. However, recruitment was much slower than expected. Consequently, recruitment was extended to 10 months (June 2017 to March 2018). During this time, a total of 894 patients (130 in the intervention arm and 764 in the control arm) were screened for eligibility.

In the control arm, all patients on the study wards were screened by research nurses for potential eligibility against the inclusion criteria, which corresponded with the AMBER care bundle eligibility criteria. Patients, however, were deemed as being eligible only after confirmation by the clinical team. Subsequently, a member of the clinical team was required to explain to patients (or their relatives) their situation of clinical uncertainty and the purpose of the study. The most common reason preventing research nurses from approaching potential participants to ask for their informed consent was uncompleted clinical tasks, for example the failure to speak with family members about a patient’s current clinical situation. For the intervention arm, the identification of potential patient participants for inclusion in the study was guided by clinical decisions on whether patients were suitable to be supported by the AMBER care bundle. Since clinical teams assessed patients on the study wards to be supported by the AMBER care bundle on their daily handover meetings, the research nurses did not repeat this process. Although a larger number of patients were assessed for eligibility in the control arm of the study, the final number of patients who were eligible (*n* = 117) is very similar to those who were eligible in the intervention arm (*n* = 103).

Table 1 presents the number of patients screened and successfully recruited at each of the study sites. Only 1.9% (*n* = 8) and 8.9% (*n* = 28) of those screened in the control arm (both sites) were eventually recruited. The screening log for control site 1 provided detailed information on the characteristics of eligible and ineligible patients, and those who consented or refused. The log reported that of the patients screened who met the first eligibility criterion (*deteriorating, clinically uncertain, and with limited reversibility*), 55 (15.1%) did not meet the second criterion (*at risk of dying during their current episode of care)*. Other sites provided only a limited range of information concerning potential recruits and control site 2 did not routinely keep a recruitment log. Recruitment rates were higher at the intervention sites, where 25.0% (*n* = 20) and 18.0% (*n* = 9) of those screened were recruited, respectively.

**Table 1:** Summary of numbers screened, excluded and recruited, by site and study arm

	Control site 1	Control site 2	Intervention site 1	Intervention site 2	Total
Screened	449	315	80	50	894
Not eligible	365	282	22	5	674
*N* (%) of those screened who were eligible	84 (18.7)	33 (10.4)	58 (72.5)	45 (90)	220 (24.6)
Reasons for non-recruitment					
Lacked capacity and no caregiver	4	2	8	6	20
Too unwell	19	0	2	6	27
Died	0	0	7	5	12
Discharged or discharge planned	13	0	6	2	21
Declined	5	2	6	6	19
Clinical review/tasks	28	1	4	8	41
No reason provided	7	0	5	3	15
*N* and % of those eligible who were recruited	8 (9.5)	28 (84.8)	20 (34.4)	9 (20)	65 (29.5)

Clinicians and research nurses at the control sites reported challenges in identifying potential patients who fulfilled the eligibility criteria, particularly regarding the risk of dying during their current episode of care. Unlike health professionals at the intervention sites, they were not trained and guided in identifying these patients, as providing this education and support may have resulted in potential contamination of patient care. Since one of our study objectives was to examine critically how the study operated under field conditions, we reviewed patient recruitment in terms of the eligibility criteria over 4 months to assess the feasibility of recruitment. As a result, a pragmatic decision was made with the trial steering group to remove the ‘risk of dying’ criterion, focusing instead on just the first AMBER care bundle eligibility criterion, i.e. patients who are deteriorating and patients whose situations are clinically uncertain, with limited reversibility. A substantial protocol amendment was obtained from the NHS research ethics committee, in addition to local research governance permissions. We planned to monitor the effect of this change on recruitment, but control site 1 did not have the capacity to implement the revised recruitment strategy by the time approvals had been obtained. Control site 2 recruited eight more participants after this change.

### Sample characteristics

Participants in both trial arms were predominantly white British and widowed, and most were either living comfortably or coping with their present level of income. The majority (64.6%, *n* = 42) of all 65 patient participants lacked mental capacity and therefore, proxy assent was required on their behalf. Control site 2, in an urban setting, had a more ethnically diverse sample profile compared to the other sites. There were differences between the trial arms (Table 2). In the control arm, most patients were men aged between 65 and 79 years with a cancer diagnosis, while in the intervention arm, the majority were women aged 80 years or older with a non-cancer diagnosis. The older age and non-cancer diagnoses of the patients in the intervention arm are likely due to the inclusion of two care-of-the-elderly wards at intervention site 1.

**Table 2:** Baseline participant characteristics by trial arm

	*N* (%) for whole sample, *N = 65*	*N* (%) for control, *N* = 36	*N* (%) for intervention, *N* = 29
Gender			
Male	33 (50.8)	22 (61.1)	11 (37.9)
Female	32 (49.2)	14 (38.9)	18 (62.1)
Age			
50–64	10 (15.4)	9 (25.0)	1 (3.5)
65–79	25 (38.5)	18 (50.0)	7 (24.1)
80+	30 (46.2)	9 (25.0)	21 (72.4)
Mean (standard deviation)	77.8 (12.3)	71.8 (10.8)	85.3 (9.7)
Disease group			
Cancer	30 (46.2)	23 (63.9)	7 (24.1)
Non-cancer	35 (53.8)	13 (36.1)	22 (75.9)
Patient had capacity			
Yes	23 (35.4)	17 (47.2)	6 (20.7)
No	42 (64.6)	19 (52.8)	23 (79.3)
Education			
Did not go to school	3 (4.6)	3 (8.3)	0
Secondary school (GCSE/O Level)	21 (32.3)	9 (25.0)	12 (41.3)
Secondary school (A Level)	15 (23.1)	6 (16.7)	9 (31.0)
Vocational qualification	4 (6.2)	2 (5.6)	2 (6.9)
University	11 (16.9)	7 (19.4)	4 (13.8)
Prefer not to say	7 (10.8)	6 (16.7)	1 (3.5)
Missing	4 (6.2)	3 (8.3)	1 (3.5)
Marital status			
Single	10 (15.4)	6 (16.7)	4 (13.8)
Widowed	26 (40.0)	9 (25.0)	17 (58.6)
Married, civil partnership, or long-term relationship	27 (41.5)	19 (52.8)	8 (27.6)
Divorced	1 (1.5)	1 (2.8)	0
Missing	1 (1.5)	1 (2.8)	0
Ethnicity			
White British	45 (69.2)	17 (47.2)	28 (96.6)
Other white	2 (3.1)	1 (2.8)	1 (3.5)
White or black African	1 (2.8)	1 (2.8)	0
White and Asian	1 (2.8)	1 (2.8)	0
Other mixed	1 (2.8)	1 (2.8)	0
Indian	7 (10.8)	7 (19.4)	0
Pakistani	1 (2.8)	1 (2.8)	0
Other Asian	4 (6.2)	4 (11.1)	0
Caribbean	1 (2.8)	1 (2.8)	0
Other black	1 (2.8)	1 (2.8)	0
Missing	1 (2.8)	1 (2.8)	0
Income			
Living comfortably at present	26 (40.0)	14 (38.9)	12 (41.4)
Coping on present income	21 (32.3)	12 (33.3)	9 (31.0)
Difficult on present income	5 (7.7)	0	5 (17.2)
Very difficult on present income	2 (3.1)	2 (5.6)	0
Prefer not to say	2 (3.1)	0	2 (6.9)
Don’t know	6 (9.2)	5 (13.9)	1 (3.5)
Missing	3 (4.6)	3 (8.3)	0

### Reasons for hospital admission

Reasons for the patient participants’ admission to hospital included shortness of breath, falls, and confusion. Out of 65 participants, 62 (95.4%) had an unplanned admission to hospital through an emergency department. They had a range of different illnesses (Table 3), and an average of 2.3 comorbidities (range 1–4) (Table 4), the most common being those associated with circulatory disorders.

**Table 3:** Number of morbidities by International Classification of Diseases, Revision 10 by study site, study arm, and total

Morbidity	Control site 1 *N = 8*	Intervention site 1 *N = 20*	Intervention site 2 *N = 9*	Control site 2 *N = 28*	Control arm *N = 36*	Intervention arm *N = 29*	Total *N = 65*
Neoplasms	7	2	1	17	24	3	27
Respiratory system	0	1	4	7	7	5	12
Mental disorders	1	11	1	3	4	12	16
Circulatory system	4	8	3	14	18	11	29
Musculoskeletal	0	4	2	4	4	6	10
Blood disorder/endocrine	4	2	1	11	15	3	18
Digestive system	3	2	0	3	6	2	8
Neurological	0	4	3	4	4	7	11
Other	0	1	2	1	1	3	4

**Table 4:** Number of morbidities by study site, study arm, and total

No of morbidities per patient	Control site 1 *N = 8*	Intervention site 1 *N = 20*	Intervention site 2 *N = 9*	Control site 2 *N = 28*	Control arm *N = 36*	Intervention arm *N = 29*	Total *N = 65*
Missing	0	1	1	0	0	2	2
1	1	9	3	7	8	12	20
2	3	5	2	11	14	7	21
3	4	4	2	5	9	6	15
4	0	1	1	5	5	2	7
Mean (standard deviation)	2.38 (0.74)	1.84 (0.96)	2.13 (1.13)	2.29 (1.05)	2.31 (0.98)	1.93 (1.00)	2.33 (1.09)

### Descriptive analyses of candidate primary outcome measures

Table 5 presents the levels of missing data and an exploratory analysis of patient primary outcomes for the IPOS subscale and the howRwe at each of the time points (mean and SD). The mean IPOS subscale score at the baseline was 13.3 (SD 4.8) in the control arm and 13.3 (SD 5.1) in the intervention arm, i.e. within the moderate range. This remained fairly consistent across the time points: 13.3 at 3–5 days (change of 0 from the baseline) and 10.3 at 10–15 days (change of − 3.0 from the baseline) in the control arm; 14.6 at 3–5 days (change of + 1.3 from the baseline) and 13.9 at 10–15 days (change of + 0.6 from the baseline) in the intervention arm. Although the howRwe scores changed across time points, it is not possible to comment on whether this is clinically significant because of the small sample size and the high rate of attrition between time points.

**Table 5:** Descriptive analysis of participant self-reported outcomes for participants who had data at baseline and 3–5 days, by study arm

Primary outcome measures		Baseline, mean (SD)	3–5 days, mean (SD)	10–15 days, mean (SD)
IPOS subscale ^5^	Control, *N* = 12	13.3 (4.8)	13.3 (3.9)	10.3 (1.2) ^1^
	Intervention, *N = 12*	13.3 (5.1)	14.6 (4.1)	13.9 (5.3) ^2^
howRwe ^6^	Control, *N = 8*	13.1 (2.5)	13.9 (2.5)	14 (2.0) ^3^
	Intervention, *N = 2*	11.5 (2.1)	12.0 (0)	11 (N/A)^4^

^1^*N* = 3

^2^*N* = 7

^3^*N* = 3

^4^*N* = 1

^5^ A higher score is worse for patients. For the subscale, seven items are scored 0–4. Possible scores range from 0 to 28

^6^ A higher score is better. Four items are scored 1–4. Possible scores range from 4 to 16

*SD* standard deviation

### Economic evaluation process

The descriptive statistics of service use showed that utilisation was within plausible ranges. Patients interviewed at 10 – 15 days, reported the use of investigations/tests and the informal care provided, but no health service use due to hospitalisation was reported between baseline and follow-up. Deriving EQ-5D index score was feasible for those who answered the questions on five dimensions (Table 6). Furthermore, we determined it was feasible to collect the data on health and social care service use, informal care provision and quality of life at baseline and at 10-15 days. Missing values in the data were not problematic (less than 9.0%). We decided not to calculate preliminary cost-effectiveness because attrition at 10-15 days reduced the number of paired samples available to twelve.

**Table 6:** Health and social care utilisation and informal care provision for the past 3 months at the baseline interview

	Control					Intervention				
	*N*	User		Utilisation		*N*	User		Utilisation	
		*n*	%	Mean	s.d.		*n*	%	Mean	s.d.
Overnight stay										
Intensive care unit	36	6	17.0	4.2	5.4	29	0	0.0	n/a	n/a
Inpatient ward	36	22	61.0	10.8	10.2	29	15	52.0	22.3	25.9
Hospice	36	3	8.0	60.0	n/a	29	0	0.0	n/a	n/a
Nursing home	36	2	6.0	3.0	n/a	29	1	3.0	76.0	n/a
Residential home	36	2	6.0	69.5	13.4	29	4	14.0	55.0	29.8
A&E	36	15	42.0	1.9	1.1	29	12	41.0	1.8	1.7
Emergency ambulance	36	15	42.0	1.9	1.3	29	13	45.0	1.5	0.7
Outpatient										
Palliative care	36	4	11.0	2.0	0.8	29	1	3.0	1.0	n/a
Radiotherapy	36	7	19.0	2.0	1.2	29	0	0.0	n/a	n/a
Oncology clinic	36	13	36.0	2.5	1.5	29	1	3.0	2.0	n/a
Other appointment	36	12	33.0	2.5	1.4	29	6	21.0	2.0	1.3
Hospital transport ambulance	36	2	6.0	12.5	16.3	29	4	14.0	7.3	5.6
GP face to face	36	28	78.0	2.7	1.6	29	25	86.0	3.4	2.6
GP on the phone	36	24	67.0	2.9	1.2	29	17	59.0	2.6	2.5
Nurse										
Marie Curie	36	4	11.0	1.3	0.5	29	1	3.0	2.0	n/a
McMillan or palliative care	36	9	25.0	3.4	2.3	29	1	3.0	1.0	n/a
Other	36	4	11.0	1.5	0.7	29	3	10.0	1.0	0.0
Palliative care or hospice at home team	36	7	19.0	3.0	2.6	29	0	0.0	n/a	n/a
Physiotherapist	36	8	22.0	2.4	0.9	29	7	24.0	2.5	1.9
Occupational therapist	36	6	17.0	2.0	1.1	29	6	21.0	2.4	1.5
Psychiatrist	36	0	0.0	n/a	n/a	29	0	0.0	n/a	n/a
Psychologist or counsellor	36	3	8.0	1.7	1.2	29	0	0.0	n/a	n/a
Spiritual care person	36	0	0.0	n/a	n/a	29	3	10.0	6.3	3.8
Social worker	36	5	14.0	3.5	4.4	29	0	0.0	n/a	n/a
Paid formal carer	36	4	11.0	90.0	0.0	29	13	45.0	20.7	24.0
Dietician	36	9	25.0	1.8	0.7	29	4	14.0	3.0	2.6
Voluntary service	36	1	3.0	0.0	n/a	29	0	0.0	n/a	n/a
Other professionals	36	4	11.0	1.0	0.0	29	2	7.0	35.5	48.8
Investigation or diagnostic tests										
Blood test	36	35	97.0	13.8	8.7	29	18	62.0	5.6	6.5
X-ray	36	28	78.0	3.3	3.8	29	13	45.0	2.7	1.1
Echocardiogram	36	9	25.0	1.5	0.5	29	5	17.0	1.0	0.0
Electrocardiogram	36	20	56.0	1.9	1.0	29	10	34.0	1.2	0.4
Ultrasound	36	17	47.0	1.5	0.8	29	3	10.0	1.5	0.7
Computed tomography scan	36	27	75.0	1.8	1.0	29	7	24.0	1.2	0.4
Magnetic resonance image	36	13	36.0	1.6	0.9	29	1	3.0	2.0	n/a
Other	36	17	47.0	4.3	6.0	29	7	24.0	1.2	0.4
Informal care (hours)										
Personal care	36	20	56.0	30.9	44.8	29	15	52.0	16.3	29.6
Help with medical procedures	36	18	50.0	8.4	5.6	29	12	41.0	5.5	9.2
Help inside the home	36	24	67.0	6.6	4.3	29	17	59.0	6.5	4.2
Help outside the home	36	25	69.0	8.3	6.9	29	17	59.0	2.3	1.4
Time spent on call	36	13	36.0	26.5	51.7	29	11	38.0	48.2	68.6
Other	36	4	11.0	4.7	2.5	29	4	14.0	7.3	9.5
EQ-5D index score	33	–	–	0.00	0.33	28	–	–	−0.08	0.14

*A&*E accident and emergency department, *GP* general practitioner, *n/a* not applicable. *s.d.* standard deviation

### Patient participant views on being involved in the study

Patient participants considered their involvement in the study positively. Only one participant, who was in the control arm, did not want to complete the study questionnaire (no reason was stated). Some stated they were happy to participate due to the positive interaction with the research nurses, for example: ‘The research coordinator is very polite and explained everything about the study’ (Con2-014). Others were motivated by a sense of altruism, believing involvement would help others and improve services: ‘If you can help others, then it’s worth doing’ (Int2-007). A number of participants also encouraged other patients to take part in the study, reiterating that their involvement would ‘help others’.

### Focus groups with health professionals

In total, we conducted four focus groups with health professionals, one at each of the four study sites. Their views focused on the following issues: (i) the eligibility criteria for the AMBER care bundle and its implications for patient eligibility in the study, (ii) considerations of study settings and processes, and (iii) the impact of the feasibility study on research nurses. Details of the participants in the four focus groups at each of the study sites are presented in Table 7. Themes and illustrative quotes are presented in Table 8.

**Table 7:** Characteristics of health professionals attending focus groups by study site

Site	Intervention site 1 *(N = 11)*	Intervention site 2 *(N = 15)*	Control site 1 *(N = 9)*	Control site 2 *(N = 11)*
Specialties	Geriatrics	Respiratory	Haematology Diabetes	Rheumatology Endocrinology
Job titles (gender)	Consultant geriatrician, ward X (F) Consultant geriatrician, ward Y (M) Ward clerk, ward Y (F) Ward sister, ward Y (F) Ward manager (F) Ward manager assistant (F) Physician associate, ward X (F) Matron, ward X (M) Nurse assistant (M) Research nurse (F) Research nurse (F)	Junior ward sister (F) Staff nurse (F) Registrar (F) Senior house office (F) Foundation Year 1 Doctor (F) Senior house office (F) Junior doctor (M) Matron (F) Palliative care clinical nurse specialist (F) Research nurse (F) Ward manager (F) Junior doctor (M) Senior house office (F) Registrar (M) Foundation Year 1 Doctor (M)	Locum senior house officer (M) Band 5 occupational therapist (F) Ward sister (F) Research nurse (F) Research practitioner (F) Matron of research (F) Staff nurse (F) Palliative care consultant (M) Senior house officer (F)	Consultant rheumatologist (M) Consultant endocrinologist (F) Physiotherapy technician (F) Research coordinator (F) Rheumatology senior house officer (F) GP senior house officer (F) Foundation Year 1 Doctor (M) Registrar rheumatologist (M) Foundation Year 1 Doctor (F)
Duration	50 minutes	49 minutes	60 minutes	65 minutes

*F* female, *M* male

**Table 8:** Themes and illustrative quotes from focus groups with health professionals

Issues	Illustrative quotes
Concerns relating to study eligibility criteria	
Subtleties in relation to the study eligibility criteria	*It’s quite* *subjective, but that’s probably good. If you put strict criteria, you might miss some. Like we were saying, it’s almost like a* *feeling* *isn’t it, that’s someone is uncertain. It’s not this metric thing. You know, otherwise like this is a marker of uncertainty. I quite like the fact that the criteria are … just that, uncertain.* Int1013-M-CONS *My reflection is I think it’s just a bad expression [the eligibility criteria]. It’s the question of defining what the ‘episode of care’ is. It’s really what’s it came down to, wasn’t it? Initially, I was told that the episode of care finishes as the patient leaves the backdoor, which really isn’t true, is it? That’s the whole point of whoever is following their care to the community, as far as a patient is concerned. I’m hoping their perception of an episode of care isn’t ‘The back door is closed, you’re in the ambulance going home and that’s it.’* Con2020-M-CONS
Professional discordance and the eligibility criteria	*I think that’s been particularly difficult in this study. I think that is why we struggled to recruit. Because what we perceived to be a patient wasn’t certain (i.e. uncertain), and this was not the view of the medical team. I think it’s either ‘We are actively treating’ or ‘End of life’ [approving hhhmms in the background from the other research nurses]. You know there’s no ‘in-between’.* Con1021-F-RN *The medics would say ‘No, they’re not’* *[about patients identified as fulfilling study eligibility criteria]. But just listening to the handovers, it was like you’d identify* *everybody* *in the ward.* Con1018-F-WS *It came to a point where we [the research nurses] had explained it [the study] and explained it again. I think it got to a point where they [the clinicians] just said: ‘No, no, they’re* *not* *eligible.’* Con1021-F-RN
Issues with the prognostication of dying	*My worry is ‘the risk of death’ can be differently interpreted. So, I think being a bit more concrete about the ‘risk of death’ would be good.* Con2020-M-CONS *Well, sometimes it’s hard predicting whether they’ll die during this admission or when they’re going home … They might not die in this admission, but they are at the end of life in the next few months.* Int2019-F-SHO
Contamination of usual care	*I’ve learned a lot from being involved in the ImproveCare study. I think it made it much more comfortable for me to go for these discussions. I think when I was earlier, pretty early in my training days, it was very difficult, when we got asked all these different questions, probably I didn’t have answers for and they kept asking why can’t we do this, why can’t we do that and I didn’t understand but then when you get a better understanding of it, if you’re comfortable in touching these subjects.* Con2019-M-REG
Study setting and study processes	
Consultant oversight of study ward	*The consultant changes every week and there’s there are five or six of them, aren’t there? So, they’re there every fifth week and you know, you happen to tell them every week about the study, remind them that the study is going on.* Con1023-M-CONS
Misinterpreting clinicians’ explanations of the study	*The daughter of the patient told me I was ‘Dr Death’ and ‘the Grim Ripper’! They were very upset about it and I think it was largely because they didn’t understand.* Con2020-M-CONS
Process of seeking consent	*You give the four-sided A4 booklet PIS [participant information sheet] to an 80 year old. It knackers them out. They say read it to me. I get halfway through and they’re falling asleep because they are so, so, sick.* Int2003-F-RN *The consent process also needs to be changed. There is nothing to say, you have to get a ‘written consent’ and I think you need to be pushing these boundaries with the ethics committees. This is why research in this specialty is not being done. You would’ve had dozens of more questionnaires completed, dozens. Why can’t when I go in to a see a patient ask: ‘Mr Smith would you mind answering some questions about your condition and how we’ve been treating you?’ ‘Yes, no problem.’ ‘Okay. Mr Smith, you do understand that you don’t have to do this?’ ‘Yes’ ‘You do understand that you can stop at any time.’ ‘Yes.’ So, I can tick those and start asking questions. You would’ve had an 80% completion rate!* Int2003-F-RN
Challenges of recruiting patients who lacked mental capacity	*The actual recruitment process was sometimes quite difficult because of what you said, the families not being here. Most of the patients we approached, we had to contact the relatives because the patients were too unwell. So being able to liaise with the families was difficult. We can’t be waiting for the families here, so we were called back about relatives that were here and popping back and asking the ward staff to tell us when they’re here. So, the practicalities were difficult.* Int1033-F-RN
Views on being involved in the feasibility study	
Mixed views on involvement in study recruitment	*It was just two members of staff who came up every day, looking. A lot of the other staff felt* *so* *uncomfortable working in this area, if we’re honest. The two who did do it, in the end, found all the end-of-life stuff really tiring, even though wasn’t strictly an ‘end-of-life’ care study. The fact is that we got a paediatric nurse and a stroke nurse, and they felt a bit out of their depth.* Con1020-F-RP *For the patients, I don’t think there was negative impact. … However, I don’t think the staff on the ward were keen.* Con1021-F-RN *I was really, really impressed about how on-board everybody was and everybody knew what AMBER was. On the whole, generally 90% of the time people were very supportive of our presence and what we needed to do.* Int1033-F-RN
Greater insight into patients’ experiences	*(The study) gave us a unique relationship with the relatives. So, in a strange way, you're in a unique position that they talk to you about things that sometimes they feel that they can't take forward with certain ward staff*. So, we are able to encourage them into having those conversations making sure that those communications were taking place with ward staff if they, the family had some anxiety or a certain query. *So, whether because we were seen as external or whether we would able to form a relationship over questionnaires being done different points, I just don't know*. Int1033-F-RN

#### Eligibility criteria and issues with prognostication

Participants were concerned about the study’s eligibility criteria, which were informed by the intervention’s eligibility conditions. At times, the discussion focused on what was understood to be clinical uncertainty. This included confusion about the middle ground between patients who were being actively treated and those at the end of life. There was evidence of disagreement between professional groups about which patients were potential cases. We observed a sentiment of a perceived disparity of power between doctors and nurses concerning how decisions were made about which patients could be approached. At control site 1, the research nurse and ward sister explained their difficulty with recruitment because clinical uncertainty was not fully understood by doctors as being a legitimate concept. Medical staff raised serious concerns that they perceived prognostication was required to confirm a patient as at risk of dying during their stay to meet the eligibility criteria. The concept of at risk was problematic to implement as an objective criterion, with perceptions of subjectivity surrounding risk and variation in interpretation of risk within and across the clusters.

There was also discussion about when an episode of care could be considered to have ended. While this was objectively on a patient’s discharge, there was a strong sentiment that the recommendations of care should be maintained from hospital to home or care home. It was not within the scope of the study to investigate this.

Although we did not ask about study contamination at the focus groups in the control sites, a small number of participants stated that merely thinking about clinical uncertainty, albeit in the absence of an intervention to guide them, had influenced their clinical practice. Participants mentioned that the study provided them with a platform to broach difficult topics, such as clinical uncertainty and advance care planning, with patients and their families.

#### Consideration of study settings and processes

Views were shared about site-level factors external to the study protocol and how they had a bearing on the success of the study. The system of consultant oversight of a ward was a critical factor to consider when setting up the study. At one of the control sites, by the time a consultant had become familiar with the study and its requirements, they had been replaced by a new consultant who needed to be introduced to the study.

Explaining the study to patients and families, with the study documentation, was challenging for some as they assumed that the primary focus was dying. This was not the case, but these comments further highlight the degree of specificity needed when training staff in study processes and interpreting the intervention’s eligibility conditions, which governed the study’s eligibility criteria.

Health professionals also reported that the consent and consultee assent process was challenging, highlighting the extensive length of the participant information sheets and the manner in which consent was sought (as required by the research ethics committee). They suggested modifications to streamline this process.

#### The impact of the feasibility study on research nurses

The emotive and complex nature of the study was discussed. At one site, we hoped to have a larger team of research nurses, given the need to screen patients daily. However, only a few research nurses felt adequately skilled to attend ward handover meetings to identify potential patients and then lead potentially distressing encounters with patients, many of whom were very unwell. Other research nurses felt that the unique focus of the study provided them with a privileged position and opportunity to develop deeper relationships with the patient participants.

## Discussion

This account of the design and execution of this feasibility cluster RCT of the AMBER care bundle provides evidence of the important methodological issues that arise in studies of interventions for patients nearing the end of life. Whilst a full trial of the AMBER care bundle is technically possible, it would not be realistic using the methods employed. This feasibility cluster RCT study was difficult to perform for a myriad of intervention-based, logistical, and methodological reasons. However, it provides vital evidence to inform future research evaluating complex interventions for patients nearing the end of life in hospital settings.

The study has several important strengths. It was a clinical trial of a complex hospital-based intervention, recruiting 65 patient participants, many whom were elderly and frail with multiple morbidities, achieving 81.3% of our recruitment target, over an extended recruitment period. Moreover, it collected data from these individuals at multiple time points. The knowledge gained from this study contributes to progressing how research can be conducted with patients near the end of life [45, 46]. Patient participants viewed involvement in the study positively and many were grateful for the opportunity to share their views and experiences. This challenges commonly held misconceptions that research among this patient population is unnecessarily intrusive [47]. Additionally, we purposefully selected four hospitals, with different specialties, serving different parts of the country, which enhances the generalisability of our findings.

### Study eligibility criteria and recruitment

Referring clinicians and research nurses require that the eligibility criteria for a clinical trial to be clear and unambiguous. In the present study, the criteria created a number of sampling challenges outlined below.

First, the AMBER care bundle eligibility conditions operated as the eligibility criteria for the feasibility trial, which referring clinicians and research nurses found confusing. Beyond a patient being identified as deteriorating, clinically unstable with limited reversibility, patients were also required to be at risk of dying during their current episode of care, despite treatment. The combined evidence from the screening logs and the views of health professionals in the focus groups highlighted that the prognostic element of the criteria was a major impediment when identifying and recruiting potential patient participants. Whilst this finding was germane to both trial arms, it was more pronounced in the control arm due to lack of training and confidence in identifying potential patients. Prognostic models vary in levels of sophistication, ranging from clinical intuition to more intricate multivariate statistical models that combine multiple factors to yield an assessment [48]. If risk of dying is to be retained as an eligibility criterion, there is a continued risk of two sampling biases being present due to the unknown (or the inconsistent) manner in which health professionals currently interpret risk. Firstly, there is the unpredictable and often unreliable identification of potential participants within and across study sites, and secondly their exclusion. The findings from the health professional focus groups and analysis of the recruitment logs suggest both biases were indeed present. Although models to enhance the identification of dying patients using prognostic models are improving for patients with cancer [49–51], there is far less consensus on methods to assess patients with non-malignant conditions, which are more common in studies of this nature [52, 53]. If similar complex interventions are to be evaluated, the subjectivity in prognostication must be avoided and greater emphasis placed on objective clinical indicators, for example, poor performance status scores, the presence and severity of cognitive impairment, weight loss, and dysphagia.

Second, equipped with the participant information sheets, clinicians were required to introduce the study to potential patient participants and its relevance to them in relation to their clinical situation. Some clinicians, especially at the control sites, reported they lacked the confidence and skills to talk openly with patients about their circumstances. This challenge was exacerbated by research nurses’ reports that while convinced that some patients identified were ideal for the study, their views were challenged by clinical colleagues who disagreed on their suitability, stating that they were unclear that they were at risk of dying during the admission. This inadvertent gatekeeping represented an important barrier to recruitment. In the clinical care of people approaching the end of life, patients value their autonomy in decision-making. This also applies to research participation, where the opportunity to help others, and to be heard, must be respected [54]. Preventing this, whether intentionally or unintentionally, may violate the ethical principle of fairness [55]. Future studies should test methods that train health professionals in conducting difficult conversations about introducing studies of this nature to potential patient participants, whilst being mindful that this training does not contaminate the study by corresponding too closely to the intervention to be tested.

Third, it was challenging to ensure homogeneity in how patients were identified at and across sites. Frequent staff turnover (notable at one site) may lead to inconsistencies (and potentially, bias) in the way potential patients are identified and recruited. It proved challenging for the research site’s principal investigators and researchers to identify whether the criteria were systematically applied and to track reasons for non-participation. Such tracking is invaluable, alerting researchers to the need to amend recruitment approaches before the study progresses too far and a vital pool of potentially eligible patients are inadvertently excluded. Related to this, accurate reporting of the number and characteristics of patient participants successfully recruited, and data on non-participants, greatly assists in the identification of possible sample bias that may compromise a study’s validity [56, 57]. We adopted the CONSORT and Transparent Reporting of Evaluations framework [58], aiming to report clearly and transparently the selection of the study sample in relation to the study’s eligibility criteria, the characteristics of participants and non-participants, and refusals. However, only one study site provided a detailed screening log that adequately met this requirement. Without this information, not only is it more challenging to identify and correct instances of misinterpretation of study eligibility criteria and to manage potential gatekeeping, but it also prevents the wider research community from understanding potential threats to the internal validity of studies examining similar issues. During the planning stage of a study, researchers need to be transparent about the resources required for the screening process and require study sites to record screening information. Since there is currently no agreed ethical standard for recording non-identifiable information within screening logs [59], training research staff, or delegated individuals, at each study site to collect minimum patient-based data may be helpful.

### Study involvement procedures

A number of important findings are evident from the process of seeking informed consent from patient participants. The participation information sheets and consent sheets were developed in concert with our patient and public involvement members and subsequently approved to the satisfaction of the research ethics committee and the UK Health Research Authority. However, we discovered they were not always well suited to their intended audience: those who were older, often frail, and very unwell. The research nurses highlighted that the documentation was lengthy, too detailed, and complex, a result of the need to include information and contingencies for those patient participants who might lose capacity during the study. Thus, some potential patient participants were discouraged from enrolling in the study. Whilst research has focused on the ethical requirements of dementia-related research, where important lessons can be learned [60], little guidance currently exists for developing study documentation for end-of-life care studies that are adequately detailed to satisfy potential participants and research ethics committees alike [61].

Future studies in this area should consider using a briefer version of the participant information sheet when first approaching potential patient participants. If they display interest in the study, then the full version of the participant information sheet could be provided to them that includes information on transparency in accord with the recently introduced General Data Protection Regulation and information on the legal basis for data-processing.

Related to this, the process of recording informed consent also requires consideration. According to the Council for International Organizations of Medical Sciences and the World Health Organization, it is permissible for researchers and research ethics committees to consider modifying consent procedures as long as they preserve as much of the informed consent process as possible to enable participants to understand the general nature of a study and to make a meaningful informed decision whether to participate [62]. The criteria for considering changes include: (i) the research would not be feasible or practicable to carry out without modification, (ii) the research has important social value, and (iii) the research poses no more than minimal risks to participants. We believe studies like ours should be permitted to test alternative methods of obtaining consent to evaluate their acceptability and utility from the perspectives of potential participants.

### Contamination of the control group

When designing this feasibility study, we deliberately made use of a cluster design to minimise the potential of study contamination [26, 27, 63] associated with the movement of health professionals acquainted with the AMBER care bundle to a control ward, which may influence the care given to patients. This represented an improvement in the design from our previous comparative evaluation of the AMBER care bundle [34]. We were also mindful not to select study sites where similar interventions were in place. This is because changes to the standard or usual care during a clinical trial could impair the validity of the study. Although control site 1 did experience a relatively frequent turnover of medical staff, to the best of our knowledge no health professionals were familiar with the AMBER care bundle from having worked elsewhere. However, at control site 2, we become aware of a change in clinical practice associated with subtleties in maturation, or naturally occurring changes [64] in health professionals’ clinical practice resulting from them becoming more familiar with the concept of clinical uncertainty, which was not accompanied by any formal training of an intervention for patient and family care. Due to the small number of participating patients in both arms of the study, it was not possible to quantify the effect of this change accurately.

### Data collection and completeness of candidate primary outcome measures

We examined whether relevant clinical outcomes can be measured using instruments that could be easily completed by unwell patients whose recovery is clinically uncertain [65]. All participants (*n* = 65) who provided consent or proxy assent successfully completed the baseline measures. Overall, the levels of missing data for self-reported outcomes and those provided by proxies were very low for both candidate primary outcome measures. However, the howRwe is a patient-completed experience measure so that it cannot be used for those who lack adequate mental capacity. This severely restricted the number of participants who were able to complete this measure. Nevertheless, the findings indicate that data collection was generally possible. We also now believe that utilising health resources for the follow-up could be replaced by accessing patients’ medical records, assuming all the patients stay on wards. The costs associated with care service use would then be obtained using unit costs for each service item and opportunity costs (e.g. a minimum wage).

### Acceptability of the study to patient participants

We have demonstrated that patient participants were generally very positive about being involved in this feasibility study. This continued for all those who remained in the trial until the second follow-up at 10–15 days. Within this study, we have refuted legitimate concerns about engaging with what could be perceived as vulnerable patient populations at the end of life [66–68]. This study, therefore, demonstrates that when ethical and pragmatic decisions are made in relation to study design, combined with highly sensitised research nurses and researchers, the voice of patients can be heard.

### Study limitations

There are a number of study limitations associated with this feasibility study beyond those already discussed. First, guided by the AMBER care bundle development team, we used heat maps to identify wards with the highest number of annual patient deaths. Consequently, we did not include wards with similar specialties across the trial arms. This resulted in a case mix of patients that was quite different between the arms. The effect of this was most pronounced with the inclusion of care-of-the-elderly wards, which skewed the age balance across the trial arms. The mean age of the participants in the intervention arm was higher than that in the control arm. Future studies should not base the selection of study wards solely on the number of deaths per ward, and should consider other important factors, for example, ward specialty, the potential for active engagement of ward staff, and the presence of principal investigators on the ward.

Second, we are mindful that this study represented a feasibility study with no requirement for a formal power calculation since effectiveness was not being evaluated. Based on available data of deaths on wards, or within 100 days of discharge, we estimated that over the period of the feasibility study, we would be able to recruit 40–45 patients in each arm. However, we recruited 65. This reduced number of patient participants has some implications for fulfilling the key objectives of the feasibility study, specifically, understanding how best to recruit, examining study participant retention, testing data collection tools for the trial and determining what would be their optimum timing in a larger trial, and examining the acceptability of the overall trial. Additionally, the relatively small number of clusters included in the study meant that we were not able to calculate the intra-cluster correlation coefficient required for a future trial. Feasibility studies adopting this trial design should consider extending the number of clusters.

Third, the main reason for the loss to follow-up was due to participants being discharged from the study wards, which was evident in both arms of the study. Since we collected data from participants only when they were on the study wards, we were not able to continue data collection after their discharge. Future similar studies should consider a design that either aims to recruit patients at an earlier point in their hospital admission or permits follow-up after discharge. A potentially more appropriate commencement point for recruitment within a hospital could be when patients are in an acute medical unit. Decisions regarding patients’ further treatment and care within a hospital often take place in the acute medical unit, and there is often clinical uncertainty at this time. This would allow data to be collected for a larger number of patient participants at the third time point (10–15 days), who would otherwise have been discharged.

## Conclusions

In recent years the number of feasibility trials conducted in palliative care has increased and they have become an important requirement for funding bodies as well as being of high value to researchers in justifying study designs (to both funders and ethics committees). However, noticeably absent from many feasibility studies reported are those that conform to the recommendation that clear feasibility objectives are in place beforehand to inform whether the study protocol is ultimately feasible [22, 69]. This feasibility study conformed to this recommendation and concluded that whilst the study was indeed technically possible, based on the challenges reported and the number of design modifications required, it would be impractical to use the protocol tested to guide a full trial of the AMBER care bundle. This study has, therefore, accomplished an important positive objective of a feasibility trial [25]: the de-risking of funding of a full clinical trial estimated to cost £1.2 million that would be unlikely to meet the necessary patient recruitment and retention rates necessary to identify a clinically meaningful outcome. Meanwhile, however, the AMBER care bundle continues to be used extensively in many hospitals and endorsed in policy [70].

We suggest that future studies attempting to conduct research among this patient population, and importantly the complex interventions designed to benefit them, should consider the following four recommendations:

1. Effective timely participant recruitment is essential, since it has a significant impact on findings. Health professionals and research nurses involved in studies of this nature, therefore, require specific training to give them the right skills and to make them feel confident in identifying and then recruiting potential patient participants. Some may feel hesitant and on occasion upset, given the focus of the study. Training should, therefore, be accompanied by regular debriefings that openly discuss instances of study-invoked distress.
2. Palliative populations are heterogeneous and have a range of disease trajectories [71]. Study populations should, therefore, reflect the real world and be feasible to study. This may, therefore, require broad [72] rather than overly specific eligibility criteria.
3. Make use of population-based retrospective hospital-based data to examine and compare patients supported by an intervention with those in a control group, adjusted for propensity matching. Similar approaches have been successfully employed to examine the quality of care received by cancer patients [73]. Areas of care would have to be specified for patients who died during their hospital stay or within 100 days of discharge, and importantly for those who survived, a central feature of the AMBER care bundle. Domains of interest might include informing family members when death was imminent, the use of validated tools to assess common symptoms (e.g. pain), prescribing drugs for anxiety, the use of bereavement support when available, length of hospital stay, preferred and actual place of death, number of hospital readmissions, and admissions to emergency departments.
4. Feasibility studies that examine methods to research complex interventions focused on clinical uncertainty and at the end of life are vital for improving the design of future trials, giving them a greater chance of being completed successfully, on time, and with the required sample size [63, 74, 75].
